# On equivalence of algebraic and finite element formulations of prestressed bar structures

**DOI:** 10.1038/s41598-025-27079-6

**Published:** 2025-12-02

**Authors:** Jan Pełczyński

**Affiliations:** Faculty of Civil Engineering, Warsaw University of Technology, Al. Armii Ludowej 16, Warsaw, 00-637 Poland

**Keywords:** Engineering, Mathematics and computing, Physics

## Abstract

The equivalence of the finite element method and the algebraic formulation of the equations of moderately thick and thin elastic frames, beams, trusses, and grillages within the Timoshenko and Euler-Bernoullie theory derived earlier by Pełczyński, Gilewski (2019) is presented. The proposed algebraic formulas lead to the same systems of algebraic equations as in the finite element method approach with the use of exact shape functions. Furthermore, in order to extend the applicability of the formulation, derivations of equations for prestressed structures are presented. The formulations are supplemented with algorithms for building selected matrices, which are crucial for performing calculations using the derived formulation. In addition to the analyses performed, the benefits of the existence of a global deformation matrix $\textbf{B}$ are shown.

## Introduction

Modern structural engineering often relies on simplified bar models in terms of linear elasticity, which remain a fundamental tool in the analysis and design of a variety of load-bearing structures. Although the finite element method (FEM) has dominated engineering practice due to its versatility and adaptability, its fundamental drawback remains the need, with some exceptions, for approximation of displacement fields and aggregation of local matrices^1,2^. In response to these limitations, direct matrix algebraic approaches have been developed in recent decades, in which the equilibrium equations of bar structures take the form of the product of three matrices, including a constitutive one with a diagonal structure^3–5^.

This approach, although well known in the context of classical lattice theory^6–8^, has gained new relevance in research on topology optimisation^4,9^, non-linear analysis and reliability assessment of structures subject to large uncertainties^10^. It has proven particularly useful in applications where computational efficiency and formal clarity are crucial – e.g. the plastic range of the analysis^11^, shakedown techniques^12^, or in calculations using convex set techniques^13,14^. The extension of this formalism to moderately thick frames within Timoshenko’s theory^5^ has opened up new possibilities in the analysis of trusses, frames, beams, grillages, both flat and spatial.

The aim of the present work is to extend the formulations presented in the Refs. 3,5 by partially taking into account the non-linearities^15^ by considering the equilibrium equations of a bar in a deformed configuration. The geometric stiffness matrix can then be written as a product of matrices in which the diagonal self-equilibrated force matrix plays a central role. This way of approaching the issue not only extends the applicability of the theory but also allows the effects of prestressing to be taken into account in a way that is consistent with the algebraic formalism of the equations. This allows the application of an algebraic approach to the analysis of prestressed bar structures, including tensegrity, which have applications not only as structural elements^16,17^, but also as the basis for the construction of modern metamaterials^18–20^.

In the method presented in the present paper, the system of equations is built for the whole structure in a single step, eliminating the need for local matrix allocation. The introduction of the equilibrium equation in variational form for admissible displacement fields allows for formal consistency with classical methods, while offering a simplified algebraic structure. The equations shown can be readily extended to flat and spatial frames and grillages within the Timoshenko and Euler-Bernoullie theory subjected to prestressing. In addition, the proposed form provides additional potential during the form-finding of tensegrity structures^21^.

This paper is organised as follows. The first Section presents a comparison of the most popular approach, the finite element method, with the algebraic equations obtained, including graphical diagrams for trusses, frames made of Euler-Bernoullie bars and frames made of Timoshenko bars. The second Section contains a description of the tensegrity structure and the derivation of the algebraic equations in matrix forms, including the geometric stiffness matrix, which contains information on the structure’s prestress. The third Section concisely describes the main benefits of the algebraic approach. The conclusions of the present study are given in the last Section. In addition, Appendix 6 presents algorithms for the construction of selected matrices.

## Comparison of the algebraic formulation with the FEM formalism

Bar theories of linear elasticity are widely used in the design and modelling of various engineering structures. Various numerical approaches are used, although the use of the finite element method, which is based on the approximation of displacement fields, has been dominant for many years^1,2^. In the literature, there are also known publications that introduce finite elements with physical shape functions, accurately describing the displacement fields of selected types of structures^22^. Due to the fact that in many papers bar structures are analysed according to the algorithm of the finite element method^23–28^, this section presents the relation between the algorithms derived previously in the work^5^ and the FEM algorithm.

The main advantage of the algebraic formulation is the absence of any approximations, which by definition are introduced in the finite element method. Moreover, it is characterised by a concise formulation in which the equations are built for the whole structure, taking into account the boundary conditions from the beginning of the analysis, thus not requiring the construction of local matrices and their aggregation to a global form. The globalisation of the equations implies a more difficult algorithmisation of the method, which, however, does not offset its advantages.

An important feature of the algebraic formulation, which complements the discussion above, is that the bar displacements are expressed exactly in terms of the nodal displacements through the deformation matrices, without assuming any particular shape function, as is typically required in finite element discretizations. This means that, for a given structure, the displacement fields along the bars are obtained analytically from the bar-end values, ensuring that internal forces and deformations are exact within the assumptions of linear elasticity and prismatic bars.

**Fig. 1:**
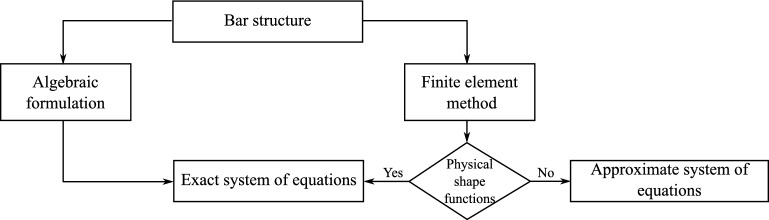
Schematic comparison of the algebraic formulation and the finite element method.

It should be emphasised that an appropriate selection of the shape function during FEM analysis can lead to an accurate system of equations for a bar structure built from both thin and medium thickness bars. In truss theory, by their very nature, the FEM equations take an exact form. However, in the theory of Euler-Bernoullie bars or Timoshenko bars, polynomial shape functions (linear or other) can be used, leading to an approximate formulation, as schematically illustrated in Fig.  1.

**Fig. 2:**
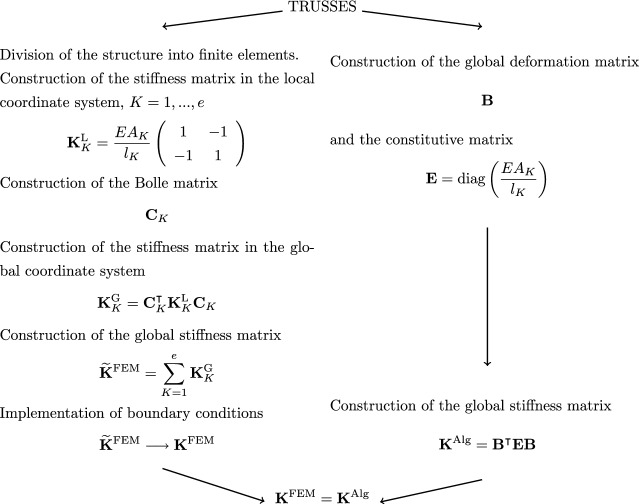
Comparison of a finite element method algorithm and an algebraic formulation for a truss.

**Fig. 3:**
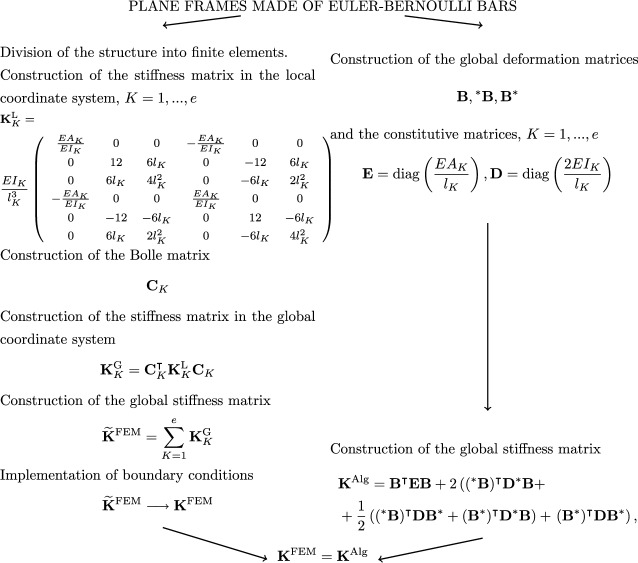
Comparison of a finite element method algorithm and an algebraic formulation for a planar frame made of Euler-Bernoulli bars.

**Fig. 4:**
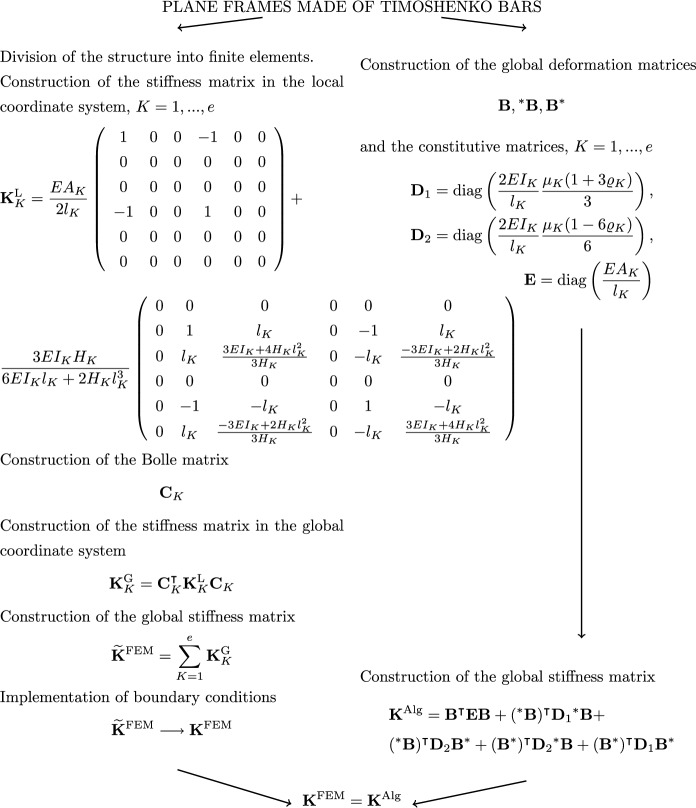
Comparison of a finite element method algorithm and an algebraic formulation for a planar frame made of Timoshenko bars.

**Fig. 5:**
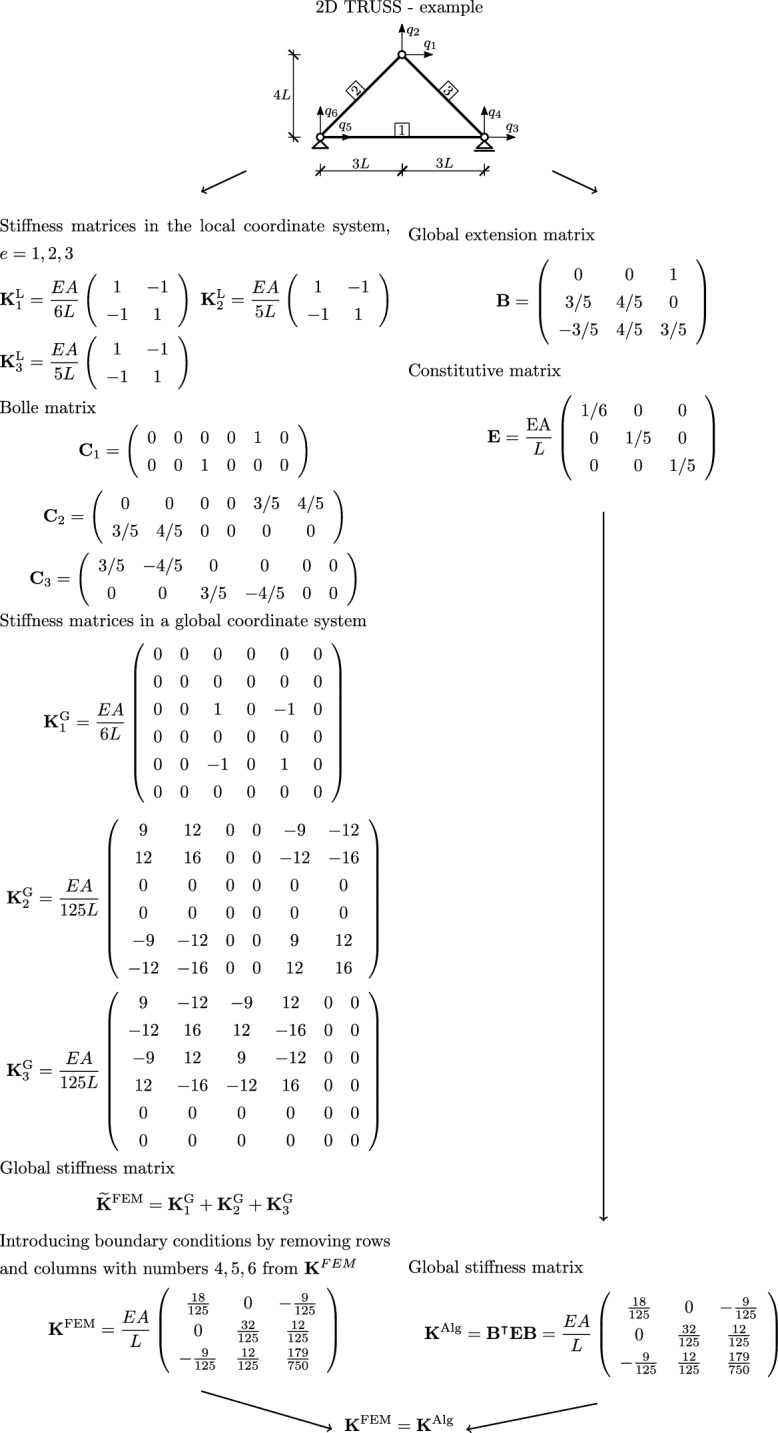
Comparison of a finite element method algorithm and an algebraic formulation for a plane truss. Computational example.

In Figs. 2, 3 and 4 diagrams are presented that descriptively show the stages of construction of the stiffness matrix resulting from FEM, with the use of physical shape functions, and the algebraic formulation for selected types of structures, that is, for trusses (see Fig.  2), 2D frames made of Euler-Bernoullie bars (see Fig.  3) and 2D frames made of Timoshenko bars (see Fig.  4). The example illustrated in Fig.  4 uses bar finite elements with physical shape functions, changing the length of the elements derived in the paper^22^ from 2*a* to *L*. The application of such functions leads to an exact formulation. Furthermore, for a better illustration of the effect of applying equations in algebraic form, a comparison for a design example of a planar truss constructed from three members is shown in Fig.  5. In the notation presented in Fig.  5, the rows of matrix $\textbf{B}$ correspond to individual truss members and contain coefficients assigned to respective degrees of freedom in equations describing the extension of these members, while $\textbf{E}$ contains the axial stiffnesses of individual members on the diagonal, which is consistent with the frequently used algebraic approach^3,5^.

Compared to the FEM formalism, the construction of the stiffness matrix in the algebraic formulation requires fewer steps and fewer intermediate objects to be defined, since the matrices are built directly for the entire structure. Although these matrices are larger, their physical interpretation is straightforward: $\textbf{B}$ directly links nodal displacements to bar extensions, while $\textbf{E}$ encodes axial stiffness. This transparency is not always apparent in the FEM procedure, where such relationships are embedded in the element shape functions and the subsequent assembly of local matrices into the global system.

The essence of the algebraic formulation presented is that the constructed stiffness matrices are identical to those obtained by the finite element method, when the finite elements with physical shape functions are used. However, their construction process does not require the approximation of displacement fields, which is present in the general FEM formulation, and there are diagonal constitutive matrices. This feature is particularly important in topology optimisation, where quadratic forms with diagonal constitutive matrices enable efficient algorithms for material distribution^4,9^. In elastic-plastic analysis, the algebraic formulation provides a clear separation of the elastic contribution in quadratic form, which simplifies the application of incremental procedures and shakedown methods^11,12^. For problems with large uncertainties, the explicit algebraic structure supports interval and probabilistic approaches, allowing rigorous bounding of the solution space^10^. Finally, in the analysis of structures with uncertain parameters, the product form of the algebraic equations with diagonal matrices naturally fits within the convex set framework, providing an effective tool for robust design and verification^13,14^.

## Algebraic formulation of tensegrity structures

**Fig. 6:**
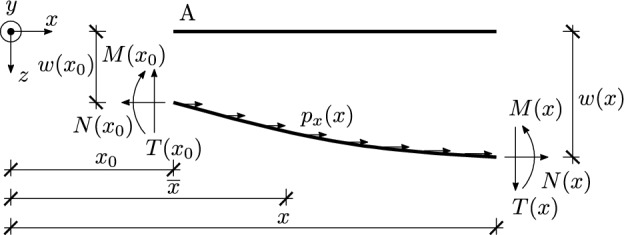
Undeformed and deformed bar in the local coordinate system *xyz* with the loading.

In the present paper, tensegrity is treated as a special case of a truss. For this reason, the derived equations are based on the truss equations. In order to introduce the prestress to the truss, realised by the self-equilibrated system of forces $S_K$^23,29,30^, let us consider the equilibrium equations of the bar in the deformed configuration. Let us consider a truss made of *e* straight and prismatic bars lying in the *X*-*Z* plane of the global coordinate system *XYZ*. $l_K$ ($K=1,2,...,e$) represents the length of the *K*-th bar and $EA_K$, $EJ_K$ its axial and flexural stiffness. The bar is subject only to a distributed load $p_{\text {x},K}(x)$ parallel to the *x* axis of the bar’s local coordinate system (see Fig. 6). The deformation of a single bar in the local Cartesian coordinate system *xyz* is described with axial displacement $u_K(x)$, transverse displacement $w_K(x)$ and rotation $\varphi _K(x)$, where *x* complies with the axis of the *K*-th bar and $0\leqslant x\leqslant l_K$.

Although the actual tensegrity structure cannot resist bending moments due to the assumption of frictionless ball joints at the nodes, the formal inclusion of bending moments in the derivation is essential for several reasons. It ensures that the presented equations remain consistent with the general framework of beam and truss equilibrium, allows the application of standard variational and matrix formulations, and preserves compatibility with earlier works on algebraic formulations of tensegrity structures^3,5^. Furthermore, it provides a clear systematic framework for relating axial forces, transverse deformations, and prestress effects, even when the contribution of bending moments is negligible. This approach not only facilitates comparison with previous studies, but also complements them, allowing potential extensions to configurations with partial flexural stiffness or different types of joints in future investigations.

The unknown internal forces: axial $N_K(x)$ and bending moment $M_K(x)$ are correlated with the strains: normal $\varepsilon _K(x)$ and curvature $\kappa _K(x)$ by

1 $$\begin{aligned} N_K(x)=EA_K\varepsilon _K(x),\quad M_K(x)=EJ_K\kappa _K(x), \end{aligned}$$

while the strains are given by

2 $$\begin{aligned} \varepsilon _K(x)=\frac{du_K}{dx},\quad \kappa _K(x)=-\frac{d\varphi _K}{dx},\quad \varphi _K(x)=\frac{dw_K}{dx}. \end{aligned}$$

The equilibrium equations in local coordinate system, written for the deformed configuration, are given by the differential equations

3 $$\begin{aligned} \frac{dN_K}{dx}+p_{\text {x},K}(x) = 0,\quad \frac{dT_K}{dx}=0,\quad T_K(x) = \frac{dM_K}{dx} + N_K(x)\frac{dw_K}{dx}. \end{aligned}$$

For prestressed structures, the axial forces can be decomposed into

4 $$\begin{aligned} N_K(x)=\tilde{N}_K(x)+S_K, \end{aligned}$$

where $\tilde{N}_K(x)$ is the force resulting from displacements of the nodes caused by external loads applied to the prestressed structure, and $S_K$ is the set of self-equilibrated forces. Let assumption $S_K \gg \tilde{N}_K(x)$ also apply. Then the additional bending moment resulting from the deformed form of the equilibrium equations and induced by the force $\tilde{N}_K(x)$ can be considered negligibly small and the Eq. (3) can be written in the form

5 $$\begin{aligned} \frac{dN_K}{dx}+p_{\text {x},K}(x) = 0,\quad \frac{dT_K}{dx}=0,\quad T_K(x) = \frac{dM_K}{dx} + S_K\frac{dw_K}{dx}. \end{aligned}$$

Then, the Eq. (5) can be written in variational form

6 $$\begin{aligned} \begin{aligned}&\int _0^{l_K}\left( N_K(x)\overline{\varepsilon }_K(x) + M_K(x)\overline{\kappa }_K(x) + S_K\varphi _K(x)\overline{\varphi }_K(x) \right) dx = \\&= \int _0^{l_K} \left( p_{\text {x},K}(x)\overline{u}_K(x)\right) dx + \left[ N_K(x)\overline{u}_K(x) + T(x)\overline{w}_K(x) - M_K(x)\overline{\varphi }_K(x)\right] _0^{l_K}. \end{aligned} \end{aligned}$$

where $[f]_0^L = f(L) - f(0)$ and the overlines $\overline{(\cdot )}$ indicate the trial fields. The relation between virtual deformations $\overline{\varepsilon }, \overline{\kappa }$ and displacements $\overline{u}, \overline{\varphi }$ is defined by (2). Further considerations assume that the kinematic boundary conditions are homogeneous with respect to preserving all given connections in the truss joints. Then the variational equilibrium equation of the truss has the form

7 $$\begin{aligned} \int \limits _\Xi \left( N_K(x)\overline{\varepsilon }_K(x) + M_K(x)\overline{\kappa }_K(x) + S_K\varphi _K(x)\overline{\varphi }_K(x) \right) d\Xi = \int \limits _\Xi p_{\text {x},K}(x)\overline{u}_K(x)d\Xi \end{aligned}$$

where $\Xi$ is the coordinate that runs through all the bars of the truss and $0 \leqslant \Xi \leqslant \sum _{K=1}^e l_K$.

The substitution of (2) to (1), and further into (5), with the use of dimensionless coordinate $\xi =x/l_K$, leads to equations which describe displacement functions $u_K(\xi )$, $w_K(\xi )$

8 $$\begin{aligned} \frac{d^2u_K}{d\xi ^2} = \frac{p_{\text {x}}(\xi )l_k^2}{EA_K}, \quad \frac{d^4w_K}{d\xi ^4} + \sigma _K^2\frac{d^2w}{d\xi ^2}= 0, \quad \sigma =i\sqrt{\frac{S_Kl_K^2}{EI_K}}, \end{aligned}$$

where *i* is the imaginary unit. The local coordinate system allows one to define the bars’ ends. According to the denotation proposed by Lewiński in Ref. 3 the values with ${\,}^*(\cdot )$ are related to the left end and the $(\cdot )^*$ to the right. The assumptions, supplemented by the assumption of zero moments at the ends of the bar, that is, $M(0)=0$ and $M(1)=0$, enable obtaining displacement fields depending on displacements of the left $({\,}^*u_K, {\,}^*w_K)$ and right $(u_K^*, w_K^*)$ bar end, which leads to the form

9 $$\begin{aligned} \begin{aligned}&w_K(\xi ) = {\,}^*w_K\left( 1-\xi \right) + w_K^*\xi , \\&u_K(\xi ) = {\,}^*u_K\left( 1-\xi \right) + u_K^*\xi . \end{aligned} \end{aligned}$$

Then the deformations (2)are given by

10 $$\begin{aligned} \varepsilon (\xi ) = \frac{1}{l_K}\Delta _K, \quad \varphi _K(\xi ) = \psi _K, \quad \kappa _K(\xi ) = 0, \end{aligned}$$

and the following quantities represent the deformations of the bar

11 $$\begin{aligned} \Delta _K=u_K^*-{\,}^*u_K, \quad \psi _K=\frac{1}{l_K}\omega _K,\quad \omega _K=w_K^*-{\,}^*w_K \end{aligned}$$

Let us consider a truss with *s* possible displacements of the nodes (called degrees of freedom), which are collected in the vector $\textbf{q}=(q_1,...,q_s)$. Then the relation between the displacements of the ends of the bars in the local coordinate system *xyz* and the displacements of the truss nodes in the global coordinate system *XYZ* is expressed by the allocation matrices.

12 $$\begin{aligned} {\,}^*u_{K}=\sum _{j=1}^{s} {\,}^*A_{Kj}^{(u)}q_{j}, \quad {\,}^*w_{K}=\sum _{j=1}^{s} {\,}^*A_{Kj}^{(w)}q_{j}, \quad u^*_{K}=\sum _{j=1}^{s} A_{Kj}^{*(u)}q_{j}, \quad w^*_{K}=\sum _{j=1}^{s} A_{Kj}^{*(w)}q_{j}. \end{aligned}$$

Therefore, relations between bars deformations (11) in the local coordinate system and displacements and $\textbf{q}$ in the global coordinate system are given by

13 $$\begin{aligned} \Delta _K=\sum _{j=1}^s B_{Kj}q_j, \quad \omega _K=\sum _{j=1}^s C_{Kj}q_j, \end{aligned}$$

or

14 $$\begin{aligned} \varvec{\Delta }=\textbf{Bq}, \quad \varvec{\omega }=\textbf{Cq}, \end{aligned}$$

where matrices $\textbf{B}=(B_{ij})$ and $\textbf{C}=(C_{ij})$ are defined by allocation matrices (12).

With the notations (13) and with the use of dimensionless coordinate $\xi$ the left-hand side of the equilibrium Eq. (7) takes the form

15 $$\begin{aligned} \int \limits _\Xi \left( N_K(x)\overline{\varepsilon }_K(x) + M_K(x)\overline{\kappa }_K(x) + S_K\varphi (x)_K\overline{\varphi }_K(x) \right) d\Xi = \sum _{K=1}^e\left( \mathbb {N}_K\overline{\Delta }_K+ \mathbb {F}_K\overline{\omega }_K \right) , \end{aligned}$$

where

16 $$\begin{aligned} \mathbb {N}_K=\int _0^1 N_K(\xi )d\xi , \quad \mathbb {F}_K=\int _0^1 S_K \varphi _K(\xi )d\xi . \end{aligned}$$

Substitution of (13) to (15) allows equality to be written

17 $$\begin{aligned} \sum _{K=1}^e\left( \mathbb {N}_K\overline{\Delta }_K+ \mathbb {F}_K\overline{\omega }_K \right) = \sum _{j=1}^s\sum _{K=1}^e\left( \overline{q}_j B_{jK}\mathbb {N}_K+ \overline{q}_j C_{jK}\mathbb {F}_K \right) \end{aligned}$$

which results in the left-hand side of the equilibrium Eq. (7) in the matrix form

18 $$\begin{aligned} \int \limits _\Xi \left( N_K(x)\overline{\varepsilon }_K(x) + M_K(x)\overline{\kappa }_K(x) + S_K\varphi _K(x)\overline{\varphi }_K(x) \right) d\Xi = \overline{\textbf{q}}^\intercal \left( \textbf{B}^\intercal \textbf{N}+ \textbf{C}^\intercal \textbf{F} \right) \end{aligned}$$

with the vectors

19 $$\begin{aligned} \textbf{N}=(\mathbb {N}_1\,\,...\,\,\mathbb {N}_e)^\intercal , \quad \textbf{F}=(\mathbb {F}_1\,\,...\,\,\mathbb {F}_e)^\intercal . \end{aligned}$$

Once the assumption of (4) has been taken into account, the Eq. (16)$_1$ can be developed into the form

20 $$\begin{aligned} \mathbb {N}_K=\int _0^1 N_K(\xi )d\xi = \int _0^1 \tilde{N}_K(\xi )d\xi + S_K = \tilde{\mathbb {N}}_K + S_K. \end{aligned}$$

Following the introduction of the designations

21 $$\begin{aligned} \tilde{\textbf{N}}=(\tilde{\mathbb {N}}_1\,\,...\,\,\tilde{\mathbb {N}}_e)^\intercal , \quad \textbf{s}=(S_1\,\,...\,\,S_e)^\intercal , \end{aligned}$$

the equilibrium Eq. (18), given that $\textbf{s}$ is an selfequilibrated system of forces, that is $\textbf{B}^\intercal \textbf{s}=\textbf{0}$, can be converted to the final form of the left-hand side of the equilibrium Eq. (7)

22 $$\begin{aligned} \int \limits _\Xi \left( N_K(x)\overline{\varepsilon }_K(x) + M_K(x)\overline{\kappa }_K(x) + S_K\varphi _K(x)\overline{\varphi }_K(x) \right) d\Xi = \overline{\textbf{q}}^\intercal \left( \textbf{B}^\intercal \tilde{\textbf{N}}+ \textbf{C}^\intercal \textbf{F} \right) . \end{aligned}$$

Right-hand side of (7) expressed with parameters ${\,}^*u_K$, ${\,}^*w_K$, $u_K^*$, $w_K^*$ and consideration of (12) leads to the form

23 $$\begin{aligned} \int \limits _\Xi p_{\text {x},K}(x)\overline{u}_K(x)d\Xi = \overline{\textbf{q}}^\intercal \textbf{Q}. \end{aligned}$$

where $\textbf{Q}=(Q_i)$ and $Q_i$ is the load work $p_{\text {x},K}$ on the virtual displacement $\overline{u}^{(i)}(x)$ corresponding to the virtual displacement field of the truss where $\overline{q}_i=0$, $\overline{q}_j \ne 0$ for $i\ne j$, $i=1,2,...,s$, $j=1,2,...,s$.

Comparison of (22) and (23) leads to the equality

24 $$\begin{aligned} \textbf{B}^\intercal \tilde{\textbf{N}}+ \textbf{C}^\intercal \textbf{F} = \textbf{Q}. \end{aligned}$$

When the bar *K*-th is loaded in the span, the internal forces $\tilde{N}_K(\xi )$ can be decomposed into parts depending on the displacements of the nodes $\textbf{q}$ and the values $N_K^0(\xi )$, which are the forces imposed by the external load applied to the statically determined truss, that is, when $q_j=0$ ($j=1,2,...,s$). Then

25 $$\begin{aligned} \tilde{N}_K(\xi ) = \frac{EA_K}{l_K}\Delta _K + \tilde{N}_K^0(\xi ). \end{aligned}$$

It is possible to proof that

26 $$\begin{aligned} \int _0^1 N_K^0(\xi )d\xi =0, \end{aligned}$$

hence the substitution of (25) into (16) leads to the constitutive equations of the frame

27 $$\begin{aligned} \tilde{\mathbb {N}}_K = \frac{EA_K}{l_K}\Delta _K, \quad \mathbb {F}_K = \frac{S_K}{l_K}\omega _K \end{aligned}$$

or in the matrix form

28 $$\begin{aligned} \tilde{\textbf{N}} = \textbf{E}\varvec{\Delta }, \quad \textbf{F} = \textbf{S}\varvec{\omega } \end{aligned}$$

with diagonal $e \times e$ constitutive matrices

29 $$\begin{aligned} \textbf{E}=\text {diag}\left( \frac{EA_K}{l_K}\right) , \quad \textbf{S}=\text {diag}\left( \frac{S_K}{l_K}\right) . \end{aligned}$$

The value of $\tilde{\mathbb {N}}_K$ is the axial force and is imposed by displacements ${\,}^*u_K$, ${\,}^*w_K$, $u_K^*$, $w_K^*$. The relation $\tilde{\mathbb {N}}_K=\tilde{N}_K(0)=\tilde{N}_K(1)=\tilde{N}_K(\xi )$ is valid with $\tilde{N}^0_K(\xi )=0$.

Substitution of (13) to (27) and then to (24) leads to a system of equations in matrix form

30 $$\begin{aligned} \textbf{K}\textbf{q} = \textbf{Q} \end{aligned}$$

with the stiffness matrix given by the formula

31 $$\begin{aligned} \textbf{K}=\textbf{B}^\intercal \textbf{E}\textbf{B} + \textbf{C}^\intercal \textbf{S}\textbf{C}. \end{aligned}$$

The spatial truss equations can be obtained by introducing the displacements in the two orthogonal planes into the relationships shown above. Thus, let the values associated with the displacements in the second plane be denoted additionally by the symbol $\widehat{(\cdot )}$. Then the geometrical relationships take the form

32 $$\begin{aligned} \varvec{\Delta }=\textbf{B} \textbf{q}, \quad \varvec{\omega }=\textbf{C} \textbf{q}, \quad \widehat{\varvec{\omega }}=\widehat{\textbf{C}} \textbf{q}, \end{aligned}$$

the equilibrium equations

33 $$\begin{aligned} \textbf{B}^\intercal \tilde{\textbf{N}}+ \textbf{C}^\intercal \textbf{F}+ \widehat{\textbf{C}}^\intercal \widehat{\textbf{F}} = \textbf{Q}, \end{aligned}$$

and constitutive relations

34 $$\begin{aligned} \tilde{\textbf{N}} = \textbf{E}\varvec{\Delta }, \quad \textbf{F} = \textbf{S}\varvec{\omega }, \quad \widehat{\textbf{F}} = \textbf{S}\widehat{\varvec{\omega }}. \end{aligned}$$

The above equations lead to a system of equations $\textbf{K}\textbf{q} = \textbf{Q}$ with the stiffness matrix given by the formula

35 $$\begin{aligned} \textbf{K}= \textbf{B}^\intercal \textbf{E}\textbf{B} + \textbf{C}^\intercal \textbf{S}\textbf{C} + \widehat{\textbf{C}}^\intercal \textbf{S}\widehat{\textbf{C}}. \end{aligned}$$

The Appendix 6 presents algorithms for the construction of the matrices $\textbf{B}$, $\textbf{C}$, $\widehat{\textbf{C}}$, as the possible workflow, the selection of directions and planes of bars, is not obvious and unambiguous. The stiffness matrix formulation presented leads to an identical matrix as the one proposed by Guest^31^. The essence of equation (35) lies in its product form, consisting of three matrices with diagonal matrices $\textbf{E}$ and $\textbf{S}$ placed in the middle of the products. The form of the first term of the Eq. (35) is well known in the literature; however, the structure of the second and third terms is original. This form does not appear in other formulations known in the literature^28,31,32^, including the finite element method. In addition to ensuring consistency and mathematical clarity, the diagonal form of the matrices in the middle of the products is of particular importance, as it enables the application of convex set-based techniques^10,13,14^ to the analysis of tensegrity structures and facilitates the acquisition of solutions to problems with uncertainties represented as convex sets.

The planar tensegrity equations presented above can also be obtained by introducing bars’ preextensions $\Delta ^{(0)}_K$ into the truss equations, which modify the geometric relations (14) to the form

36 $$\begin{aligned} \varvec{\Delta }=\textbf{B}\textbf{q} + \varvec{\Delta }^{(0)}. \end{aligned}$$

The initial extension of *K*-th bar is given by the formula $\Delta ^{(0)}_K=N^{(0)}_K l^{(0)}_K / (E A^{(0)}_K)$, in which $l^{(0)}_K$ and $A^{(0)}_K$ are the length and cross-sectional area of the bar before prestressing, respectively, and $N^{(0)}_K$ are the self-equilibrated forces induced by the initial tension. The preextension can be represented in a matrix form $\varvec{\Delta }^{(0)} = \left( \textbf{E}^{(0)}\right) ^{-1}\textbf{N}^{(0)}$. In the prestress phase, the bar extends and its cross-section decreases, so $l_K = l^{(0)}_K + \Delta l^{(0)}_K$ and $A_K = A^{(0)}_K - \Delta A^{(0)}_K$.

Then the constitutive relations take the form of

37 $$\begin{aligned} \textbf{N} = \textbf{E}\varvec{\Delta } = \textbf{E}\textbf{B}\textbf{q} + \textbf{E}\varvec{\Delta }^{(0)} = \textbf{E}\textbf{B}\textbf{q} + \textbf{E}\left( \textbf{E}^{(0)}\right) ^{-1}\textbf{N}^{(0)}. \end{aligned}$$

Let the increments of the extensions and cross-sectional areas be small, that is, $\Delta l^{(0)}_K \ll l^{(0)}_K$ and ${\Delta A^{(0)}_K \ll A^{(0)}_K}$. Then the product $\textbf{E}\left( \textbf{E}^{(0)}\right) ^{-1} = \textbf{I}$, and $\textbf{I}$ is a unitary matrix. That is, the forces in the bars after prestress can be approximated by the values $\textbf{N} = \textbf{E}\textbf{B}\textbf{q} + \textbf{N}^{(0)}$. Performing transformations consistent with those presented in the Ref. 33 leads to equilibrium equations of the form

38 $$\begin{aligned} \textbf{B}^\intercal \textbf{N} + \textbf{C}^\intercal \textbf{S}\textbf{C} = \textbf{Q}, \end{aligned}$$

since $\textbf{N}^{(0)}$ is a self-equivalent system, so $\textbf{B}^\intercal \textbf{N}^{(0)} = \textbf{0}$. The matrix $\textbf{S} = \text {diag}\left( S_K/l_K\right)$, and $\textbf{C}=(C_{ij})$ is defined by the slopes of the bars $\psi _K=\sum _{j=1}^sC_{Kj}q_j/l_K$. The final equation is identical to that derived in the variational approach.

**Fig. 7:**
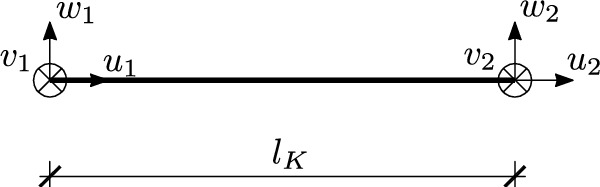
Finite element of a truss bar.

Another way leading to the tensegrity stiffness matrix is to use the finite element method formalism^1,2^, aggregating the linear stiffness matrices of the individual finite elements of the truss bar

39 $$\begin{aligned} \textbf{K}_{\text {l}K} = \frac{EA_K}{l_K} \left( \begin{array}{cc} 1 & -1 \\ -1 & 1 \end{array} \right) , \quad K=1,...,e, \end{aligned}$$

transformed from the local *xyz* to the global *XYZ* coordinate system using generalized Bolle matrices $\textbf{C}_K$

40 $$\begin{aligned} \textbf{K}_\text {L} = \sum \limits _{K=1}^e \textbf{C}_K^\intercal \textbf{K}_{\text {L}K} \textbf{C}_K, \end{aligned}$$

where *e* is the number of finite elements. The stiffness matrix of the *K*-th element is derived by assuming a vector of local degrees of freedom of the form $\textbf{q}_{\text {L}K}=(u_1\,\,u_2)^\intercal$, while $u_1$ and $u_2$ are defined on the Fig.  7.

The introduction of prestressing forces $S_K$ into the truss finite elements and the partial inclusion of non-linearities associated with the equilibrium equations written for the deformed shape are performed by adding the geometric stiffness matrix $\textbf{K}_\text {G}$ to the linear stiffness matrix $\textbf{K}_\text {L}$^23,24,27,28,31^. Geometric stiffness matrices in local coordinate systems

**Fig. 8:**
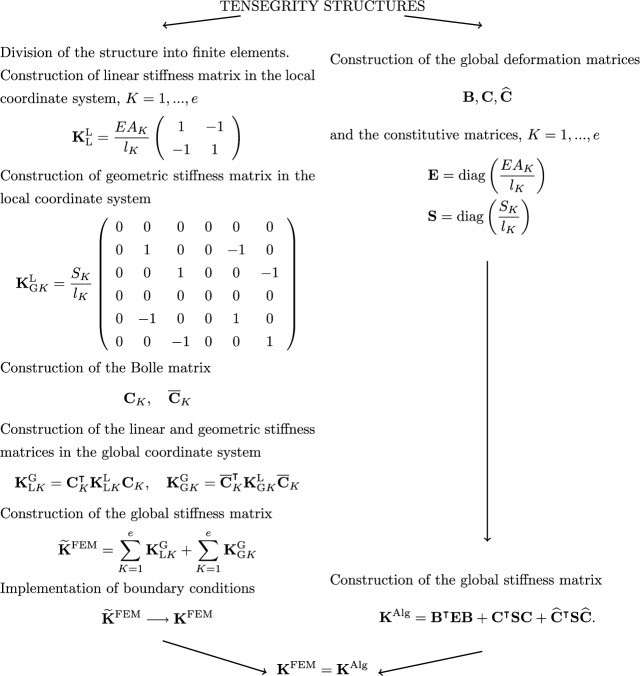
Comparison of a finite element method algorithm and an algebraic formulation for a tensegrity structure.

41 $$\begin{aligned} \textbf{K}_{\text {G}K} = \frac{S_K}{l_K} \left( \begin{array}{cccccc} 0 & 0 & 0 & 0 & 0 & 0 \\ 0 & 1 & 0 & 0 & -1 & 0 \\ 0 & 0 & 1 & 0 & 0 & -1 \\ 0 & 0 & 0 & 0 & 0 & 0 \\ 0 & -1 & 0 & 0 & 1 & 0 \\ 0 & 0 & -1 & 0 & 0 & 1 \\ \end{array} \right) , \quad K=1,...,e, \end{aligned}$$

are derived by assuming a vector of local degrees of freedom of the form $\textbf{q}_{\text {G}K}=(u_1\,\,v_1\,\,w_1\,\,u_2\,\,v_2\,\,w_2)^\intercal$, which components are defined on the Fig.  7. The use of generalised Bolle matrices $\overline{\textbf{C}}_K$ allows the geometric stiffness matrix to be aggregated and written in a global coordinate system

42 $$\begin{aligned} \textbf{K}_\text {G} = \sum \limits _{K=1}^e \overline{\textbf{C}}_K^\intercal \textbf{K}_{\text {G}K} \overline{\textbf{C}}_K. \end{aligned}$$

All of the methods presented above lead to identical forms of the tensegrity stiffness matrix as further illustrated in Fig.  8. It should be noted that the most common method for constructing the geometric stiffness matrix is FEM, although attempts to obtain it by other means are known to be made^32,34,35^. The algebraic approach presented in the present paper and presented in Ref. 33 is the author’s solution.

## Discussion

A fundamental advantage of the algebraic approach in the analysis of prestressed structures, compared to the classical finite element method, is the possibility of using the global extension matrix $\textbf{B}$. This matrix provides a direct tool for describing the deformation of members relative to node displacements and allows the systems of equilibrium equations to be written in a compact and formally explicit form. With this construction, it is possible to

- Directly identify the self-stress – self-equilibrated systems of forces can be detected by analysing the $\textbf{B}$ matrix kernels, which finds application in, among other things, the design of prestress in tensegrity structuress;
- Identify the structural mechanisms – the eigenvalues of the $\textbf{B}^\intercal \textbf{B}$ product corresponding to zero indicate the presence of mechanisms, both rigid body motions and additional mechanisms, which makes it possible to unambiguously determine the degree of static and kinematic determinability of the system;
- Maintain a concise notation and reduce algorithmic complexity – unlike FEA, which requires the creation of matrices in local coordinate systems and their assembly into a global system, the algebraic approach allows the global matrix to be written directly. This allows significant simplification of the numerical code, especially in environments that support symbolic calculus;
- Avoid approximation – an important difference from FEM is the complete elimination of the approximation of displacement fields. This means that the resulting systems of equations are accurate from the beginning, without the need for shape function approximations, making this approach particularly valuable in the analysis of structures with unusual geometries or under non-standard loading conditions.

The search for a self-equilibrated system of forces $S_K$ and finite and infinitesimal rigid movements can be carried out by a singular value decomposition (SVD) of the matrix $\textbf{B}\in \mathbb {R}^{e\times s}$ of the form

43 $$\begin{aligned} \textbf{B}=\textbf{U}\varvec{\Sigma }\textbf{V}^\intercal , \end{aligned}$$

where $\textbf{U}\in \mathbb {R}^{e\times e}$ and $\textbf{V}\in \mathbb {R}^{s\times s}$ are orthogonal matrices and $\varvec{\Sigma }\in \mathbb {R}^{e\times s}$ is a diagonal matrix. The elements on the diagonal $\varvec{\Sigma }$ are called the singular values of the matrix $\textbf{B}$.

Let us consider the products

44 $$\begin{aligned} \begin{aligned}&\textbf{B}\textbf{B}^\intercal = \textbf{U}\varvec{\Sigma }\textbf{V}^\intercal \textbf{V}\varvec{\Sigma }^\intercal \textbf{U}^\intercal = \textbf{U}\varvec{\Sigma }\varvec{\Sigma }^\intercal \textbf{U}^\intercal ,\\&\textbf{B}^\intercal \textbf{B} = \textbf{V}\varvec{\Sigma }^\intercal \textbf{U}^\intercal \textbf{U}\varvec{\Sigma }\textbf{V}^\intercal = \textbf{V}\varvec{\Sigma }^\intercal \varvec{\Sigma }\textbf{V}^\intercal . \end{aligned} \end{aligned}$$

The right-hand sides of the above relations describe the distribution of the matrix with respect to the eigenvalues. The columns of $\textbf{V}$ are the eigenvectors of $\textbf{B}^\intercal \textbf{B}$, the columns of $\textbf{U}$ are the eigenvectors of $\textbf{B}\textbf{B}^\intercal$, and the non-zero elements of $\varvec{\Sigma }$ are the square roots of the non-zero eigenvalues of $\textbf{B}^\intercal \textbf{B}$ or $\textbf{B}\textbf{B}^\intercal$.

Let us introduce the designations $\textbf{M} = \varvec{\Sigma }\varvec{\Sigma }^\intercal$ and $\textbf{L} = \varvec{\Sigma }^\intercal \varvec{\Sigma }$. Then $\textbf{B}\textbf{B}^\intercal = \textbf{U}\varvec{\Sigma }\textbf{U}^\intercal$ and ${\textbf{B}^\intercal \textbf{B} = \textbf{V}\textbf{L}\textbf{V}^\intercal }$. The decomposition of the SVD can be done by solving two eigenproblems

45 $$\begin{aligned} \left( \textbf{B}\textbf{B}^\intercal - \mu _i \textbf{I}\right) \textbf{u}^{(i)}=\textbf{0} \quad (i=1,...,e), \quad \left( \textbf{B}^\intercal \textbf{B} - \lambda _j \textbf{I}\right) \textbf{v}^{(j)}=\textbf{0} \quad (j=1,...,s). \end{aligned}$$

Then

46 $$\begin{aligned} \textbf{M} = \left( \begin{array}{cccc} \mu _1 & 0 & \cdots & 0 \\ 0 & \mu _2 & \cdots & 0 \\ \vdots & \vdots & \ddots & \vdots \\ 0 & 0 & \cdots & \mu _e \\ \end{array} \right) , \quad \textbf{L} = \left( \begin{array}{cccc} \lambda _1 & 0 & \cdots & 0 \\ 0 & \lambda _2 & \cdots & 0 \\ \vdots & \vdots & \ddots & \vdots \\ 0 & 0 & \cdots & \lambda _s \\ \end{array} \right) , \end{aligned}$$

where $\mu _i$, $\lambda _j$ are the eigenvalues and $\textbf{u}^{(i)}$, $\textbf{v}^{(j)}$ the eigenvectors of $\textbf{B}\textbf{B}^\intercal$ and $\textbf{B}^\intercal \textbf{B}$ respectively. The occurrence of zero values in the matrix $\textbf{M}$ indicates the presence of a self-stress state, while in the matrix $\textbf{L}$ there is the presence of a mechanism that may be finite or infinite.

The decomposition of the extension matrix $\textbf{B}$ according to singular values allows certain properties of a given truss to be determined. The decomposition involves the product $\textbf{B}\textbf{B}^\intercal \equiv \textbf{D}$, which is a component of the symmetric equilibrium equation of the truss $\textbf{D}\textbf{N} = \textbf{B}\textbf{Q}$. The eigenvectors of $\textbf{B}\textbf{B}^\intercal$, corresponding to the zero eigenvalues and denoted according to the SVD by $\textbf{v}^{(j)}$, realise the relation $\textbf{D}\textbf{v}^{(j)}=\textbf{0}$, i.e. they satisfy the equilibrium equations identically. The force distributions in the members of the truss, described by $\textbf{v}^{(j)}$, are self-equilibrated force systems, in the literature often referred to as self-stress^23,29,36,37^.

If the matrix $\textbf{B}$ describes an unsupported truss, then the number of zero eigenvalues of the product $\textbf{B}^\intercal \textbf{B}$, also found in the SVD, corresponds to the number of possible rigid motions of this structure and the number of possible additional mechanisms, and the corresponding eigenvectors describe these motions. There are three rigid motions in the case of a plane truss and six in a spatial structure. The additional mechanisms (finite or infinitezimal) are described by the eigenvectors corresponding to the additional zero eigenvalues of the product $\textbf{B}^\intercal \textbf{B}$^23,29,36^.

Identification of whether the additional mechanism is finite or infinite involves analysis of the stiffness matrix supplemented by a geometric stiffness matrix $\textbf{K}_\text {G}$, which depends on the system of self-equilibrium forces. If the matrix $(\textbf{K}_\text {L}+\textbf{K}_\text {G})$ of the supported truss is positively defined, this means that the corresponding mechanism is infinitezimal. Let us note the similarity of the problem of decomposition into eigenvalues of the matrix $\textbf{B}^\intercal \textbf{B}$ oraz $\textbf{K}_\text {L}=\textbf{B}^\intercal \textbf{E}\textbf{B}$. These problems are identical when the matrix $\textbf{E}$ is unitary, leading to the interesting observation that the eventual occurrence of infinitezymal mechanisms does not depend on the material properties of the lattice.

It should be noted that when a tensegrity structure has one infinitesimal mechanism and one self-equilibrated system of forces, then its description involves a quadratic extension matrix $\textbf{B}\in \mathbb {R}^{s\times s}$. This situation applies to all tensegrity structures where the support has been chosen to eliminate only rigid motions^38^.

## Conclusions

In the present paper, the equivalence of the formulation of the finite element method and the algebraic formulation of the equations of moderately thick and thin elastic frames, beams, trusses and grillages within the Timoshenko and Euler-Bernoullie theory derived earlier in the paper^5^ is presented. The proposed algebraic formulations lead to the same systems of algebraic equations as in the finite element method with exact shape functions. The considerations are supported by diagrams showing schematic formulations for trusses, frames made of Euler-Bernoullie bars, and frames made of Timoshenko bars. It should be emphasised that the use of exact physical shape functions in standard finite element practice is not common, and approximate displacement shape functions are employed more frequently. The algebraic formulation bypasses these approximations by providing a global representation of displacements, enabling a more immediate connection between nodal degrees of freedom and bar extensions. This also reduces the number of preparatory steps and intermediate objects required compared to FEM, while keeping the physical interpretation of the resulting matrices transparent.

Furthermore, in order to extend the applicability of the formulation, derivations of algebraic equations for tensegrity structures are presented. The equations shown can be readily extended to flat and spatial frames and grillages within the Timoshenko and Euler-Bernoullie theory subjected to prestressing. The derivations are supplemented with algorithms for building selected matrices, which are crucial for performing calculations using the derived formulation.

In addition to the analyses performed, an important advantage of the algebraic approach is shown, which is the existence of a global deformation matrix $\textbf{B}$ (referred to as the “extension matrix” in many publications). Its analysis allows one to determine the value and distribution of the self-equilibrated system of self-stress forces and infinitesimal rigid motions, which are the essence of the tensegrity structures.

It should be emphasised that the advantages of the approach indicated in Ref. 5 remain in force and are as follows:

- *Direct formulation without approximation*: The method does not require assumptions about the displacement fields, ensuring an exact representation of the bar extensions for the given structural configuration.
- *Natural definition of deformation measures*: The deformation measures of the truss and frame correspond directly to the physical quantities (axial extension, rotation), facilitating clear interpretation and post-processing.
- *Minimal data requirements*: Node numbering is not needed; defining the number of bars and degrees of freedom is sufficient, reducing setup complexity.
- *Algorithmic efficiency*: The approach is easy to implement, especially using symbolic computation tools (e.g. Wolfram Mathematica or Maple) and allows straightforward generation of global matrices for entire structures.
- *Extension to tensegrity structures*: The generalisation of previous algebraic methods^3,5^ enables the direct analysis of prestressed structures, broadening the applicability of the theory.
- *Suitability for advanced applications*: The product form of the equations with diagonal elasticity and initial stress matrices enables the use of convex set-based techniques and supports applications such as topology optimisation and uncertainty analysis.
- *Educational value*: The clarity of the algebraic formulation makes it very suitable for teaching concepts of structural mechanics and prestress effects.

## Figures and Tables

**Algorithm 1:**
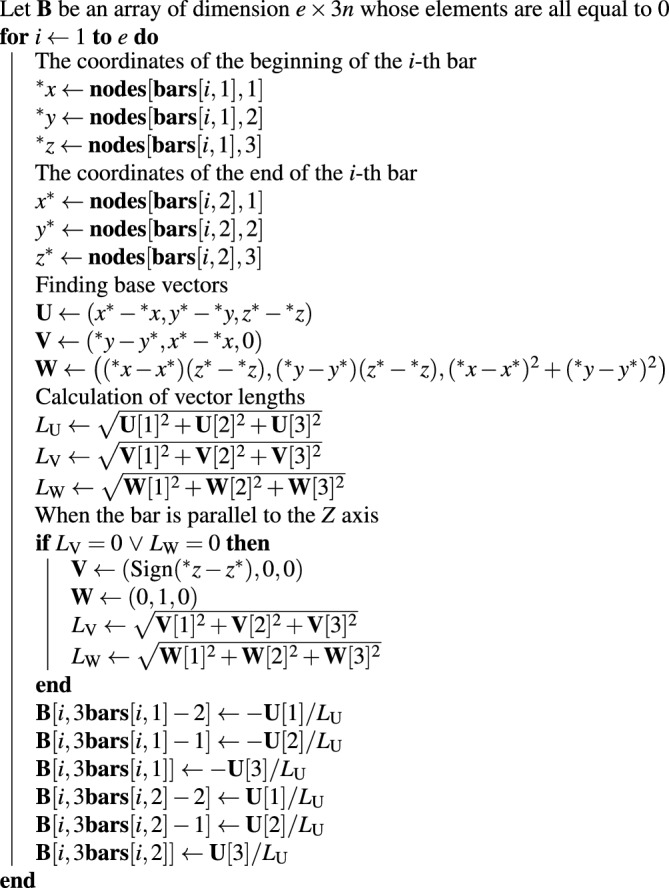
Construction of $\textbf{B}$ matrices.

**Algorithm 2:**
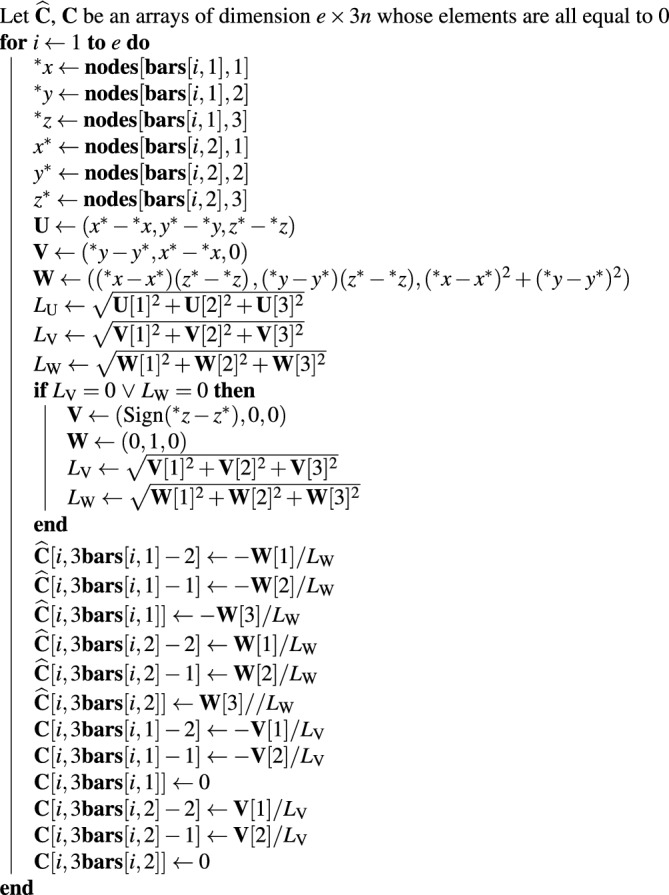
Construction of $\widehat{\textbf{C}}$ and $\textbf{C}$ matrices.

## Data Availability

All data generated or analysed during this study are included in this published article.

## Algorithms for building selected matrices for trusses

Algorithms 1 and 2 in this Appendix show how to build the $\textbf{B}$, $\widehat{\textbf{C}}$ i $\textbf{C}$ matrices for trusses, but they can easily be generalised to other types of bar structure. The matrices are constructed under the assumption that the global and local degrees of freedom have directions and rotations consistent with the axes of the global *XYZ* and local *xyz* coordinate systems, respectively. After rotation from the local *xyz* system to the *XYZ* system, the rod is rotated about the *x* axis so that the *y* axis lies in the *X*-*Y* plane and the projection direction of the *z* axis in the *X*-*Z* plane is consistent with *Z*. The given algorithms do not consider boundary conditions.

Let be given an *n*-element array of $\textbf{nodes}$ containing the coordinates of the nodes of the structure

47 $$\begin{aligned} \textbf{nodes} = \left( \begin{array}{c} (X_1,Y_1,Z_1) \\ (X_2,Y_2,Z_2) \\ \vdots \\ (X_n,Y_n,Z_n) \\ \end{array} \right) , \end{aligned}$$

and *e*-element array $\textbf{bars}$ containing pairs (start node number, end node number) and thus defining the position of each bar.

The three orthogonal vectors $\textbf{U}$, $\textbf{V}$, $\textbf{W}$ occurring in the algorithms presented below have the following interpretation: $\textbf{U}$ is a vector with a direction parallel to the bar and a direction from the start node to the end node; $\textbf{V}$ is a vector parallel to the *X*-*Y* plane, perpendicular to $\textbf{U}$, directed so that the direction of the projection of vector $\textbf{W}$ on the *X*-*Z* plane is consistent with the direction of the *Z* axis; $\textbf{W}$ is a vector perpendicular to $\textbf{U}$ and $\textbf{V}$.
